# Intervertebral disk-like biphasic scaffold—demineralized bone matrix cylinder and poly(polycaprolactone triol malate)—for interbody spine fusion

**DOI:** 10.1177/2041731412454420

**Published:** 2012-07-23

**Authors:** Li Jin, Yuqing Wan, Adam L Shimer, Francis H Shen, Xudong J Li

**Affiliations:** Department of Orthopedic Surgery, University of Virginia Health System, Charlottesville, VA, USA

**Keywords:** Interbody fusion, tissue engineering, bone matrix, poly(polycaprolactone triol malate), bone graft

## Abstract

Interbody fusion is an established procedure to preserve disk height and anterior fusion, but fusion with autografts, allografts, and metallic cages has its endogenous shortcomings. The objective of this study is to investigate whether a biphasic scaffold model, the native demineralized bone matrix cylinder in conjunction with degradable biomaterial poly(polycaprolactone triol malate), can be employed as a biological graft for interbody fusion. The poly(polycaprolactone triol malate) was synthesized by polycondensing malic acid and polycaprolactone and then the concentric sheet of poly(polycaprolactone triol malate) was fabricated into the demineralized bone matrix cylinder derived from rabbit femurs. Rabbit chondrocytes were loaded onto the three-dimensional constructs with 1-day in vitro culture and implanted into the subcutaneous dorsal pocket of nude mice. The chondrocytes/scaffold constructs are approximately two folds bigger than the scaffold-alone constructs after 12 weeks of implantation. X-ray and micro-computed tomography imaging showed endochondral bone formation in the chondrocytes/scaffold constructs as early as 4 weeks and showed that the bone intensity increased over time. Histological staining confirmed the above observation. By week 8, lamellar bone tissues were formed inside the demineralized bone matrix cylinder. In addition, the compression biomechanical test showed that the chondrocytes/scaffold constructs produced a significant higher compressive strength compared to the scaffold group. These results demonstrated that the inner-phase poly(polycaprolactone triol malate) degraded over time and was replaced by new bone in an in vivo environment.

## Introduction

Since Cloward began anterior interbody fusion of the cervical spine with autogenous iliac bone grafts, anterior cervical discectomy and fusion has been established as one of the operative procedures to distract the disk distance, enlarge the neural foramen, and obtain anterior interbody fusion.^1^ Bone grafts are usually used for stimulating the fusion of the two vertebrae. Tricortical iliac crest autograft (bone from the same patient) serves as a gold standard for interbody fusion, since the graft has strong osteoconductive and osteoinductive potentials for bone remodeling^2^; however, the harvesting from the iliac crest can be associated with short- and long-term morbidity in up to 22% of the cases.^3^ This donor site morbidity has fueled the search for various forms of allograft materials as alternatives for cervical interbody fusion. Yet, allograft incorporates more slowly and has risk of disease transmission and immunogenicity.^4^ Nowadays, metallic cage devices have gained increasing popularity; however, titanium, one of the major components, has an elasticity modulus six times larger than the cortical bone.^5^ The altered biomechanics of the cervical spine from insertion of a metal scaffold may lead to unfavorable effects. In addition, some shortcomings of metallic cages, such as cage migration, subsidence, adjacent level degeneration, stenotic myelopathy, and nonunion, have already been reported.^6–8^ Long-term assessment of these cage devices, especially in multilevel applications, has been less than satisfactory with the incidence of revision procedures increasing over the last several years. Tissue engineering is an attractive technique for bone replacement,^9–11^ which has several advantages, including no donor site morbidity, decreased operating time, and conformation to the bone shape.

Most synthesized bone scaffolds have good osteoconductive but not osteoinductive property. Some natural materials such as collagen, hyaluronic acid, and chitosan have the regeneration qualification but with inadequate mechanical properties.^12^ In our previous study, we have demonstrated that poly(polycaprolactone triol malate) (PPCLM) (obtained by polycondensation of polycaprolactone (PCL) and malic acid) had good biocompatibility in a foreign body response in vivo assay.^13^ Incorporation of demineralized bone matrix (DBM) into the scaffold enhanced the compressive strength compared with PPCLM alone. In addition, the tensile stress of the DBM/PPCLM scaffold was 50-fold greater than that of PPCLM alone.

In the present study, we proposed a biphasic scaffold model for the interbody fusion by combining decalcified allograft and biodegradable materials. The inner phase of the scaffold is composed of a biomaterial based on PPCLM, oriented in a concentric sheet and seeded with chondrocytes. The outer phase of the scaffold is composed of DBM derived from rabbit femurs, which enclose a high content of type I collagen. The PPCLM and DBM were fabricated into a three-dimensional (3D) scaffold. Thus, the DBM/PPCLM constructs not only stimulate the intervertebral disk structure to fuse the vertebrae but also have both osteoconductive and osteoinductive properties. To explore the usefulness of this scaffold in interbody fusion, we characterized the cell proliferation, bone formation, and mechanical properties of the construct by subcutaneous implantation of the constructs in nude mice.

## Material and methods

### Synthesis of PPCLM

PPCLM was synthesized as previously described by our group.^13^ Briefly, the malic acid (0.1 mol/L) and PCL (0.05 mol/L) chemicals were mixed and melted at 140°C with a constant stirring of nitrogen gas. After melting, the prepolymer was formed by lowering the temperature to 120°C for 1 h under nitrogen gas stirring. Then the PPCLM prepolymer was dissolved in tetrahydrofuran to form a 25 wt% solution, followed by addition of sieved salt. The resulting slurry was cast into poly(tetrafluoroethylene) dishes. After evaporating for 24 h, the molds were then transferred into an oven for postpolymerization. The salt in the composites was washed out by successive incubations in deionized water (Milli-Q, Billerica, MA) every 12 h for 96 h. The porous sponge-like foams were freeze-dried and stored in desiccators for future use.

### Preparation of DBM

DBM cylinder tubes were prepared following our published method.^14^ The femurs were harvested from New Zealand white rabbits, and dissected free from connective tissues and washed with sterilized water. The femurs were then extracted with a mixture of chloroform and methanol (1:1 ratio) for 1.5 h followed by demineralization with hydrochloric acid for 15–18 h at 2°C and were sequentially washed with deionized water, 2 M CaCl_2_ at 2°C for 1 h, 0.5 M ethylenediaminetetraacetic acid (EDTA) for 1 h, 8 M LiCl for 1 h, and, finally, deionized water at 55°C. The DBM tubes (about 8 mm in diameter and 5 mm in height) were then equilibrated with Dulbecco’s modified Eagle’s medium (DMEM) containing 100 units/mL of penicillin and 100 mg/mL of streptomycin for 1 h at 37°C, and were stored at −70°C until use.

### Fabrication of DBM/PPCLM scaffold

The DBM/PPCLM scaffolds were fabricated by introducing the PPCLM scaffold into the DBM tubes.^13^ Briefly, the long stripes (2 mm × 5 mm × 40 mm) of PPCLMs were rolled over and inserted into the DBM tubes to form the DBM/PPCLM scaffold.

### Isolation of chondrocytes

The chondrocytes were isolated with enzyme digestion following previous published method.^14^ The rib cartilages were dissected from the male New Zealand rabbits. The cartilages were cut into pieces and digested for 3–6 h with 0.1% collagenase type II (Worthington, Freehold, NJ) at 37°C and then were resuspended in DMEM with 4.5 g/L glucose, 10% fetal bovine serum, 50 mg/L sodium ascorbate, 10 mm (4-(2-hydroxyethyl)-1-piperazineethanesulfonic acid (HEPES), 100 units/mL penicillin, and 100 mg/mL streptomycin. The primary chondrocytes were cultured in a humidified incubator with 5% CO_2_ for 5–7 days. The cells were marinated in subconfluent, and the medium was changed every other day. The second passage of cells was used for experiments.

### Culture of chondrocytes on the DBM/PPCLM scaffold

Rabbit chondrocytes were cultured onto scaffolds as previously described.^13,15^ The strips of PPCLM were presoaked with 75% ethanol for 2 h and washed with an excess amount of phosphate-buffered saline (PBS) in a sterile condition. Approximately 5 × 10^6^ cells/mL of chondrocytes were seeded onto the strips and were then incubated for 4 h prior to the addition of the culture medium. The complexes were cultured for another 24 h, and then, the rolled PPCLM sheets were inserted into the DBM scaffolds for subcutaneous implantation.

### Subcutaneous implantation of scaffold

Animal protocols were approved by the University of Virginia Animal Care and Use Committee. The athymic NCr-nu/nu mice were purchased from Jackson’s laboratory. The scaffolds and chondrocytes/scaffolds were subcutaneously implanted in the dorsal space of the nude mice. Briefly, a 15-mm incision was cut in the dorsal skin of the mice between the scapulars using a size 15 scalpel blade, and a scaffold was embedded into the pocket. The samples were harvested at 1, 2, 4, 6, 8, and 12 weeks after implantation and fixed with 4% paraformaldehyde. Each group has four implants at each time point.

### Two-dimensional radiographs and 3D in vivo μCT imaging

At 4, 6, 8, and 12 weeks after implantation, 2D radiographs of the specimen (four samples of each time point) were taken to qualitatively assess bone formation. For the quantitative evaluation of bone formation, in vivo micro-computed tomography (µCT) was performed at the same time points.^16^ The mice were anesthetized by isoflurane and placed in an in vivo µCT system (Viva-CT, Switzerland). Following reconstruction of the 2D slices, an appropriate threshold was chosen. From 2D slices, 3D images were created using a feldkamp back projection algorithm (Exxim Corp, Pleasanton, CA). The 3D image was sectioned with Image J software (National Institutes of Health, Bethesda, MD), with one sagittal image per pixel. The volume of the bone tissue was selected and defined as the region of interest (ROI). Quantitative data of bone mass and volume were normalized to the data from the DBM at the same specimen.

### Histological studies

The implants were embedded into paraffin and cut into 7-µm-thick sections. The sections were subjected to standard hematoxylin and eosin (H&E), and Safranin-O staining as previously described.^13,17^ The images were captured with a Nikon Eclipse E600 microscope (Nikon, Melville, NY).

### Compression test

The compression properties were tested as our published method.^13^ To test this tensile property of the scaffold, the implants were cut into a rectangular shape and the longitudinal tensile stress was measured on an Instron 5544 mechanical tester equipped with a 500-N load cell (Instron, Canton, MA). The samples were compressed at a rate of 10 mm/min and stopped at 60% strain. All samples were tested in triplicate, and data are presented as mean ± standard deviation (SD).

### Statistical analysis

The data are presented as mean ± SD at a significance level of p < 0.05. The statistical differences between groups were calculated using a Student’s t-test.

## Results

### Chondrocytes growth in the subcutaneous implant

**Figure 1.**
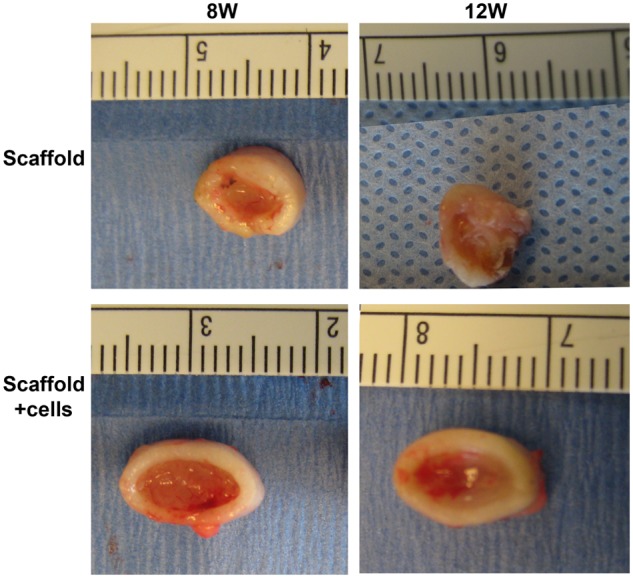
Morphology of implanted constructs. The PPCLM with or without chondrocytes were rolled and inserted into DBM rings followed by subcutaneous implantation into back skin space of nude mice. The specimens were harvested at different time points. The graph shows the representative image of scaffold with or without chondrocytes at weeks 8 and 12. Some of the scaffold constructs were collapsed in scaffold-alone group. PPCLM: poly(polycaprolactone triol malate); DBM: demineralized bone matrix.

We have successfully demonstrated that chondrocytes can survive in the PPCLM/DBM complex in vitro.^13^ We hypothesized that this cell-integrated scaffold can form bone-like tissue in an in vivo environment. To address this question, we implanted the constructs subcutaneously into the dorsal space of the nude mice. There were no significant size difference between the chondrocytes/scaffold and scaffold-alone construct at the first 2 weeks after subcutaneous implantation. However, the sizes of chondrocytes bearing constructs started to distinguish from the scaffold-alone constructs from the fourth week postoperatively, and size differences became gradually obvious until the samples were harvested at 12 weeks. The sizes of the chondrocytes/scaffold constructs were about two folds bigger than the scaffold-alone constructs at postoperative 12 weeks as showed by the morphology (Figure 1). Some of the constructs were collapsed in the scaffold-alone group at weeks 8 and 12.

### Bone formation of chondrocytes/scaffold

**Figure 2.**
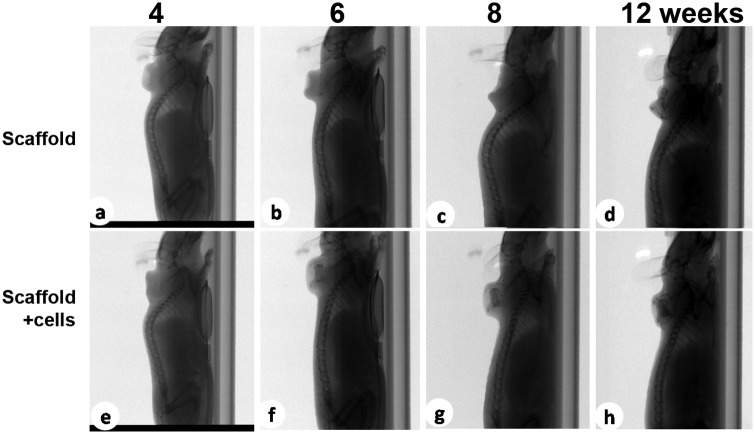
X-ray images showed de novo new bone formation in chondrocytes/scaffold constructs. At 4, 6, 8, and 12 weeks after implantation, two-dimensional radiographs of the specimen were taken to qualitatively assess bone regeneration.

**Figure 3.**
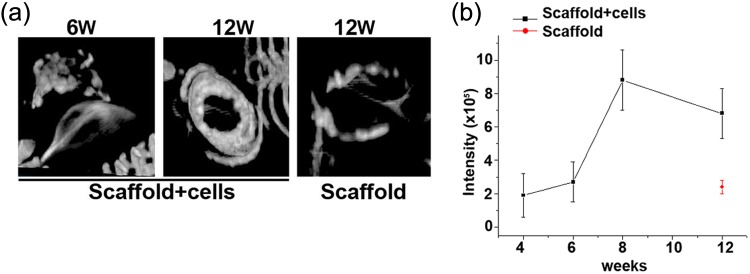
Representative µCT 3D images showed well-formed bone tube in (a) scaffold + cells group but not in scaffold-alone group. (b) Quantitative bone intensity data were shown in line graph, n = 4, p < 0.05. µCT: micro-computed tomography; 3D: three-dimensional.

We further tested the capability of bone formation of the constructs in this model. The radiographs of the specimens were obtained at each time point. As shown in the radiographs (Figure 2), there were no de novo bone formation in both constructs at week 2, and the bone tissue was detected in the cell-bearing group as early as week 4. The intensity of bones developed denser as the longer of the implantation time, and reached with the maximum intensity at week 12, the longest time point observed (Figures 2 and 3). Of note, the implants of the scaffold-alone construct started to partially collapse at week 6, and there was much less bone formation at week 12. In contrast to the very thin and disorganized bone formation in the scaffold-alone group, µCT 3D reconstruction images exhibited the well-formed bone tube in the chondrocytes/scaffold implants (Figure 3, n = 4, p < 0.05). These observations were consistent with the morphology study.

### Histological morphology of the chondrocytes/scaffold implants

**Figure 4.**
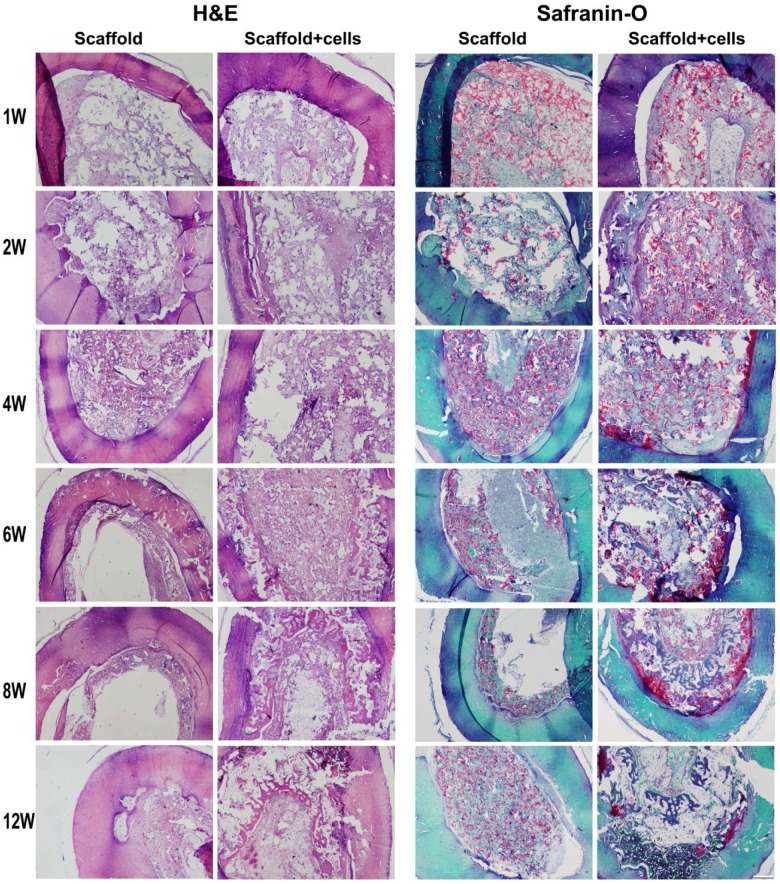
Histological studies confirm new bone formation in scaffold with chondrocytes group. At 1, 2, 4, 6, 8, and 12 weeks after implantation, the specimens were fixed and embedded and followed by H&E (left) and Safranin-O staining (right). Magnification was at 2× objective lens. In Safranin-O staining, the bone tissue was stained with green, the PPCLM scaffold with light red, and the endochondral bone formation with dark red. Scale bar = 500 µm. PPCLM: poly(polycaprolactone triol malate); H&E: hematoxylin and eosin.

**Figure 5.**
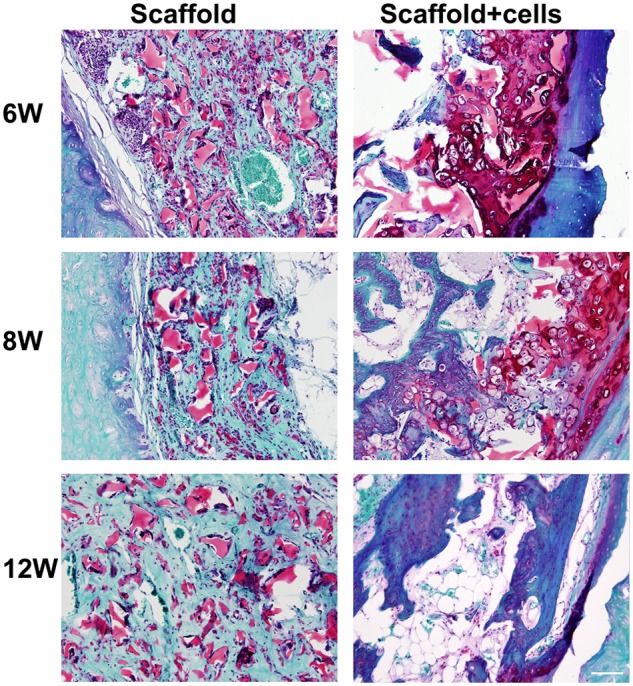
Safranin-O staining showed endochondral bone formation at 6, 8 and 12 weeks with higher magnification. Magnification was at 10× objective lens. Scale bar = 100 µm. PPCLM: poly(polycaprolactone triol malate); H&E: hematoxylin and eosin.

Histology with H&E staining (left panel) and Safranin-O (right panel) is shown in Figure 4. In the early time point at 1 week postoperatively, layers of PPCLM structure could be seen in the scaffold-alone group, but different layers of PPCLM in chondrocytes/scaffold group have already mingled with each other and chondrocytes distributed evenly in the PPCLM polymers. At 2 weeks postoperatively, both groups lost the PPCLM concentric layers with PPCLM degradation and cell ingrowths and/or proliferation. There was more cellularity in the chondrocytes/scaffold group than the scaffold-alone group. At 4 weeks, endochondral bone formed in the chondrocytes/scaffold group, which was evidenced by the dark red color observed with Safranin-O staining in the junction of the chondrocytes/PPCLM with DBM. As the implantation time increases, lamellar bone tissues are formed in the chondrocytes/PPCLM constructs within the DBM cylinder in addition to further endochondral bone formation (Figure 5), while in the scaffold-alone group, only fibrous tissue is formed along the inside rim of the DBM cylinder with void space in the center (Figures 4 and 5).

### Mechanical properties of the chondrocytes/scaffold implants

**Figure 6.**
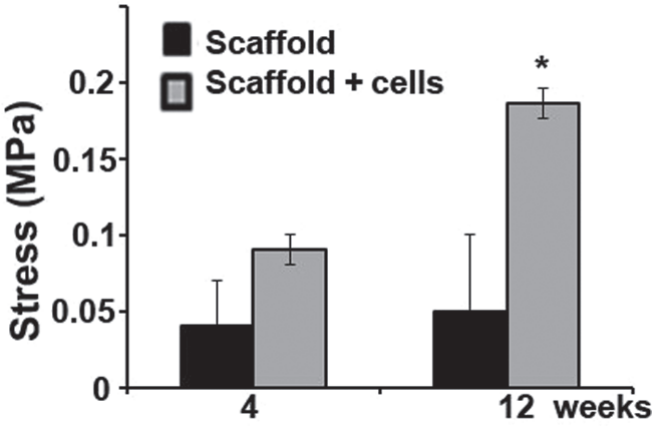
Scaffolds with chondrocytes showed higher mechanical stress compared to scaffold-alone group. The specimens were harvested at 4 and 12 weeks after implantation for compression test. The scaffold with chondrocytes groups showed significant higher strength in scaffold/cells group than scaffold-alone group.

The biomechanical property is critical for bone function. We, therefore, tested the biomechanical features of these implants. The compression test showed that chondrocyte-integrated scaffolds produce a significantly higher compressive strength (fourfolds) compared to the scaffold-alone ones (Figure 6) at week 12, while there was small difference in compression strength (about twofolds) at week 4 between the two groups. In addition, the chondrocytes/scaffold constructs showed a linear increase in the strain–stress curve.

## Discussion

As the metallic cages have many shortcomings, various biodegradable cages have been developed with tissue engineering approaches.^18–22^ Herein, we have developed a biphasic scaffold using a native DBM as an outer layer and the PPCLM as an inner layer loaded with chondrocytes in the shape of intervertebral disk for interbody fusion. We demonstrated that the chondrocytes proliferated on the biphasic engineered constructs and endochondral new bone formation developed 4 weeks postoperatively in scaffold with chondrocytes in subcutaneous environment.

The biphasic scaffold in the current study contains an outer DBM cylinder to simulate the native bone tissue, which has several advantages^14,23,24^: (a) the DBM cylinder is a natural biomaterial with superior biocompatibility properties to most of the synthetic scaffolds developed thus far. Unlike most commercial DBM, the DBM cylinder provides structural strength; (b) DBM contains proteins and growth factors that necessitate for chondrogenesis and osteoinductivity. As we know, external growth factor(s) appliance has been well reported in spine fusion. Anterior cervical fusion with recombinant human bone morphogenic protein-2 (rhBMP-2) has an estimated 40% greater risk of adverse events with rhBMP-2 in the early postoperative period, including life-threatening events. After anterior interbody lumbar fusion, rates of implant displacement, subsidence, infection, urogenital events, and retrograde ejaculation were higher after using rhBMP-2 than controls.^25^ The DBM has different combination of growth factors/noncollagenous proteins in physiological levels, while no aforementioned side effects associated with high concentration of rhBMP-2 were reported; (c) the DBM cylinder has the potential to be partially decalcified to provide the sufficient mechanical competence according to different biomechanical requirement during bone formation; and (d) It does not evoke any appreciable local foreign body immunogenic reaction as antigenic surface structure of bone is destroyed during demineralization.

Our results showed that chondrocytes/scaffold implants have much more bone formation (Figures 2 to 5) and higher compression stress compared to the scaffold-alone implants (Figure 6), and this suggested that the PPCLM supported the chondrocyte growth and the chondrocytes and DBM together sustained the new bone formation. Histological studies showed that new bone formed in the center and periphery of the chondrocytes/scaffold constructs and that the endochondral formation was observed in the junction of PPCLM and DBM in the early stage. All these results supported that DBM has the osteoconductive, osteoinductive, and osteointegrity properties that are essential for bone tissue engineering,^26^ and that cells, either chondrocytes or stem cells, are critical for solid bone formation. Cells may serve as the targets of noncollagenous protein and growth factors released from the DBM. One of the shortcomings of this study is the lack of a chondrocytes/PPCLM/nonactive DBM control and a fibroblasts/PPCLM/active DBM control to define the role of active DBM and chondrocytes. For the future interbody fusion animal study, we will have both control groups to characterize the roles of chondrocytes and/or DBM in bone formation. We believe that chondrocytes/PPCLM and active DBM have synergic effects on the bone formation, which deserves further investigation.

One of the fundamental features of scaffolds is to support adherence and proliferation of cells. In this biphasic scaffold, the inner phase was built by polycondensing the PCL and malic acid. The PCL polymer has been widely used in bone, cartilage, cardiac, vascular, and skin tissue engineering. The degraded products of PCL are neutral in pH and thus do not alter the local environment.^27^ Malic acid–derived polymers are also used in tissue engineering as well as drug delivery.^28^ In addition, the intermediate product of malic acid is mammalian tricarboxylic acid, which is completely degraded into carbon dioxide and water. Thus, we had hypothesized that PPCLM produced by combination of PCL and malic acid was suitable for chondrocytes proliferating. Indeed, our previous in vitro data showed that chondrocytes could adhere and proliferate on both the surface and inside of the scaffolds.^13^ Consistently, in the current study, we demonstrated that chondrocytes proliferated in the scaffold as the size of the chondrocytes/scaffold constructs is much bigger than the scaffold-alone constructs after 12 weeks of implantation (Figure 1). More importantly, the chondrocytes participate in the endochondral bone formation as revealed by the histological studies (Figures 4 and 5). The bone formation was also confirmed by X-ray (Figure 2) and µCT analysis (Figure 3). The bone density observed by radiographs is not likely resulting from DBM since the µCT demonstrated well-formed bone tubes in chondrocytes/scaffold implants in contrast to the thin and disorganized bone formation in the scaffold-alone implants (Figure 3). Although there are many benefits to the use of bioresorbable implants, there has historically been a concern about the potential for aseptic inflammatory wear debris generated during implant resorption.^29,30^ In the histological sections, we did not observe any detectable necrosis area and inflammatory responses in both scaffold-alone and chondrocytes/scaffold constructs. Safranin-O staining showed that PPCLM degradation was observed since 1 week postoperatively, and chondrocytes speeded up the process, which was evidenced by loss of layered PPCLM structure in the chondrocytes/scaffold constructs versus the blurred PPCLM layers in the scaffold-alone constructs 1 week postoperatively. All these results suggest that the DBM/PPCLM is suitable for chondrocytes proliferation and osteogenesis, which is the base for bone tissue engineering.

Certain limitations existed with the current technique and require further investigation before this approach can be applied to interbody fusion in vivo. First, the interface between the DBM and PPCLM remains a potential weak area. µCT showed clear spaces between DBM and PPCLM/chondrocytes (Figure 3). Further biomechanical characterization of this zone is needed. Cancellous bone might serve a good bridge between the cortical DBM and PPCLM. Second, the vascularization of the constructs needs to be considered. Third, to keep sufficient biomechanical properties, the decalcification time length or decalcification percentage of DBM should be analyzed. Fourth, the osteoinductive capacity of DBM can be affected by storage, processing, and sterilization methods and can vary from donor to donor, thus different batches may have different potencies because of the wide variety of donors used to supply the graft.^31^ Fifth, the tissue engineering design still uses a two-step approach, in vitro culture and then in vivo implantation. In the future interbody fusion in vivo experiment, we will use autograft bone marrow aspiration from either vertebral body or iliac crest loaded on PPCLM/DBM construct directly. Despite these limitations, the use of a DBM/PPCLM biphasic scaffold appears promising and worthy of further investigation.

## Conclusion

Altogether, this study demonstrated that the biphasic scaffold with the native DBM as outer layer and the biodegradable PPCLM as inner phase, which is in the shape of an intervertebral disk, is a promising candidate for interbody fusion as evidenced by abundant endochondral bone formation and increased biomechanical properties in a subcutaneous implantation model.
